# Efficacy of a novel modified double-scope endoscopic submucosal dissection technique for large rectal lesions

**DOI:** 10.1055/a-2882-8393

**Published:** 2026-06-24

**Authors:** Yutaka Eto, Taku Yamagata, Yoshihide Kanno, Tomohiro Shimada, Kei Ito

**Affiliations:** 1 Gastroenterology 506803 Public Interest Incorporated Foundation Sendai City Medical CenterSendaiJapan

The effectiveness of traction devices during endoscopic submucosal dissection (ESD) is limited for lesions near the anus due to the narrow, confined space. 1​ While double-scope ESD (dsESD) is an effective traction method for gastric lesions, 2​ its application in the rectal region is hindered by severe frictional interference between the two scopes and the tight anal passage, which restricts maneuverability.

To address this, we developed a modified dsESD technique using a newly developed thin therapeutic endoscope (EG-840TP; Fujifilm, Tokyo, Japan; 7.9 mm in outer diameter, 3.2-mm working channel) as the main scope and an ultrathin endoscope (EG-840N; Fujifilm; 5.8 mm in outer diameter) as the assistant scope. By utilizing thin scopes for both roles, frictional interference is minimized, allowing greater maneuverability in the confined anorectal space. Our setup includes an optimized room layout and a conventional injection needle fixed to the outer rim of the assistant scope ( Fig 1 ). The assistant scope functions as a versatile “surgeon’s hand,” providing not only flexible, three-dimensional traction on demand but also optimal visualization by displacing haustral folds and facilitating precise hemostasis through lesion elevation.

**Fig. 1:**
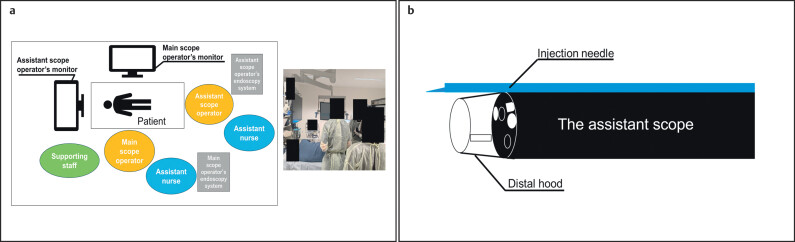
( **a** ) Schematic of the endoscopic operating room layout. The main operator stood behind the patient, while the assistant was positioned at the foot side. Each was supported by a scrub nurse, with monitors placed directly in front of them. This arrangement ensures an ergonomic environment and minimizes physical interference between the two endoscopes in the confined anorectal space. ( **b** ) The assistant scope (EG-840N) with an externally attached injection needle. To prioritize the working channel for continuous traction using grasping forceps, a conventional injection needle was fixed to the outer rim of the assistant scope. This modification also avoids the low injection efficiency associated with ultra-thin needles typically used for transnasal channels, ensuring rapid and effective submucosal lifting.

The accompanying video ( Video 1 ) demonstrates the technical efficacy of this modified dsESD technique for an 80-mm rectal lesion ( Fig. 2 ). Despite its large size, resection was successfully completed within 70 minutes without significant adverse events. Modified dsESD could be a safe and practical option for large rectal lesions.

**Video 1** Modified double-scope endoscopic submucosal dissection is an innovative technique that provides not only the desired three-dimensional traction but also excels in visualization, hemostasis, and lesion retrieval across various procedural steps.


**Fig. 2:**
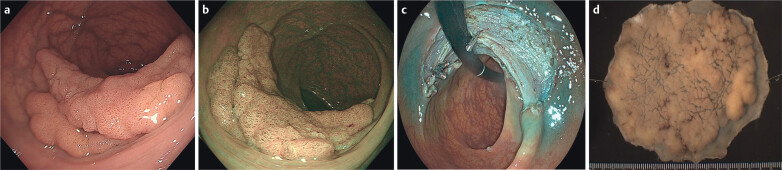
Endoscopic and pathological findings of the lesion. ( **a** ) White-light imaging shows a large LST-G located from Rs to Ra. ( **b** ) Narrow-band imaging of the lesion. ( **c** ) An endoscopic view of the ulcer bed after endoscopic resection, showing evidence of clipping on a perforating artery to ensure complete hemostasis. ( **d** ) The resected specimen measured 83×72 mm, with a lesion size of 70×63 mm. LST-G, granular-type laterally spreading tumor.

Endoscopy_UCTN_Code_TTT_1AQ_2AD_3AD

## Notice

This article was changed according to the erratum on July 20, 2026.

## Erratum

In the above-mentioned article the affiliation of all authors have been unified.The correct affiliation for all authors is: Gastroenterology, Public Interest Incorporated Foundation Sendai City Medical Center, Sendai, Japan. This was corrected in the online version on July 20, 2026.

## References

[1] Sakamoto N Taro O Hideaki R Endoscopic submucosal dissection of colorectal tumors using a traction device called the S-O clip Gastrointest Endosc 2017 59 1514 1523

[2] Yoshitaka T Masaki S Toru Y Outcomes of patients with early gastric cancer who underwent double endoscopic intraluminal surgery Surg Endosc 2016 30 178 183 25829066 10.1007/s00464-015-4179-9

