# Correction: Efficacy of a novel modified double-scope endoscopic submucosal dissection technique for large rectal lesions

**DOI:** 10.1055/a-2915-4794

**Published:** 2026-08-17

**Authors:** Yutaka Eto, Taku Yamagata, Yoshihide Kanno, Tomohiro Shimada, Kei Ito

**Affiliations:** 1 Gastroenterology 506803 Public Interest Incorporated Foundation Sendai City Medical CenterSendaiJapan



In the above-mentioned article the affiliation of all authors have been unified.The correct affiliation for all authors is: Gastroenterology, Public Interest Incorporated Foundation Sendai City Medical Center, Sendai, Japan. This was corrected in the online version on July 20, 2026.

